# Plant organic nitrogen nutrition: costs, benefits, and carbon use efficiency

**DOI:** 10.1111/nph.20285

**Published:** 2024-11-15

**Authors:** Laura Tünnermann, Camila Aguetoni Cambui, Oskar Franklin, Patrizia Merkel, Torgny Näsholm, Regina Gratz

**Affiliations:** ^1^ Department of Forest Genetics and Plant Physiology, Umeå Plant Science Centre (UPSC) Swedish University of Agricultural Sciences 90183 Umeå Sweden; ^2^ International Institute for Applied Systems Analysis Schlossplatz 1 Laxenburg A‐2361 Austria; ^3^ Department of Forest Ecology and Management Swedish University of Agricultural Sciences 90183 Umeå Sweden

**Keywords:** amino acids, *Arabidopsis thaliana*, carbon use efficiency, glutamine, organic nitrogen, root hair

## Abstract

- Differences in soil mobility and assimilation costs between organic and inorganic nitrogen (N) compounds would hypothetically induce plant phenotypic plasticity to optimize acquisition of, and performance on, the different N forms. Here we evaluated this hypothesis experimentally and theoretically.
- We grew Arabidopsis in split‐root setups combined with stable isotope labelling to study uptake and distribution of carbon (C) and N from l‐glutamine (l‐gln) and NO_3_^−^ and assessed the effect of the N source on biomass partitioning and carbon use efficiency (CUE).
- Analyses of stable isotopes showed that 40–48% of C acquired from l‐gln resided in plants, contributing 7–8% to total C of both shoots and roots. Plants grown on l‐gln exhibited increased root mass fraction and root hair length and a significantly lower N uptake rate per unit root biomass but displayed significantly enhanced CUE.
- Our data suggests that organic N nutrition is linked to a particular phenotype with extensive growth of roots and root hairs that optimizes for uptake of less mobile N forms. Increased CUE and lower N uptake per unit root growth may be key facets linked to the organic N phenotype.

## Introduction

Plants have evolved a range of adaptions for optimizing acquisition of mineral nutrients. Thus, plants experiencing low‐nitrogen (N) availability are characterized by a high‐root mass fraction, increased root branching and increased root surface area through an extensive production of root hairs, and in relevant cases, symbiotic interactions with mycorrhizal fungi. These features of N‐starved plants are well known from both old (Brouwer, 1962; Ågren & Ingestad, 1987) and more recent (Hermans *et al*., 2006) studies and molecular cues underpinning such plant responses have been described (Kiba & Krapp, 2016). Based on data from 77 studies and 129 species, Reynolds & D'Antonio (1996) observed that in the majority of the cases, the root biomass ratio increased with decreased nitrogen availability. It has been assumed that such phenotypic characteristics will increase the fitness of plants in low‐N environments through enhancing the ability to acquire the limiting resource – N (Brouwer, 1962). An increase in root hair density and/or ‐length is reported in a range of studies for nutrients that are relatively immobile in soil such as phosphorus and potassium (Gahoonia *et al*., 1997; Gahoonia & Nielsen, 1998; Bates & Lynch, 2001; Jungk, 2001; Bienert *et al*., 2021), but also for nutrients with higher mobility like inorganic N when occurring at low concentrations (Bhat *et al*., 1979; Foehse & Jungk, 1983; Ewens & Leigh, 1985; Saengwilai *et al*., 2021).

Plant N acquisition is mainly governed by two processes: diffusion (movement of N molecules through the soil water driven by a concentration gradient) and mass flow (transport together with soil water) (Nye, 1977; Tinker & Nye, 2000; McMurtrie & Näsholm, 2018). With decreasing N supply rates, concentrations of N in the soil solution decreases and hence the relative contribution of mass flow decreases. Consequently, plant responses to low‐N supply should be aimed towards optimization for N acquisition via diffusion and this is mainly accomplished through an increase in root surface area. A model describing plant optimization for N acquisition (McMurtrie & Näsholm, 2018) points to the possibility that mass flow is enhanced when the internal spacing of roots (i.e. the mean distance between roots of the same plant) is large, and when the total root surface area is low. Thus, optimization of mass flow‐driven N acquisition will predictably lead to lower N acquisition via diffusion. This suggests a trade‐off between plant optimization for diffusive and mass flow‐mediated N acquisition.

Plant acquisition of organic N should therefore primarily be governed by diffusion while acquisition of inorganic N, in particular, NO_3_^−^, should be governed by mass flow.

From the above one may conclude that for both low‐N supply and for a dominance of organic N, plant fitness is linked to characteristics that optimizes N acquisition via diffusion, and hence phenotypic shifts associated with low‐N availability should overlap with those related to organic N nutrition.

In nonmycorrhizal plants, the abundance and length of root hairs are pivotal for the total root surface area (Jungk, 2001; Smith & De Smet, 2012). As discussed above, root hair growth is highly responsive to the supply of immobile nutrients such as phosphate and potassium. Following the same logic, we can infer that root hair growth should also be responsive to immobile N forms. Root and root hair proliferation are dependent on photosynthetically derived carbohydrates but the actual costs in terms of energy and carbon (C) is strongly dependent on the source of N acquired by roots. Thus the biochemical cost for assimilation of different N forms varies and is substantially higher for NO_3_^−^ compared to NH_4_^+^ (Bloom *et al*., 2003). The difference is even greater comparing NO_3_^−^ and organic N such as the amino acids glutamine and arginine (Zerihun *et al*., 1998; Franklin *et al*., 2017). Here, the difference originates both from the lower energetic requirements for reduction and assimilation of N but also from the extra C derived from uptake of organic N. A model based on these differences in biochemical costs of assimilation predicts a significant increase in root mass fraction linked to organic N nutrition (Franklin *et al*., 2017). This would provide a feed‐forward mechanism by which the lower costs for assimilation and the C bonus from organic N uptake enables a larger root surface investment that, in turn, enhances organic N nutrition.

Carbon use efficiency (CUE), the ratio of photosynthesis to respiration, is a critical factor for the global carbon budget and a key parameter in global vegetation models. It is well known that plant CUE is influenced by nutrient, in particular N, availability (Vicca *et al*., 2012) but to what extent plant use of organic or inorganic N may affect plant CUE has not been investigated. However, the above‐described differences in C costs pertaining to uptake and assimilation of different N forms would theoretically also influence plant CUE. Analysing the potential impact of organic vs inorganic N nutrition on plant CUE may hence provide important information for the development of new global C models.

Here, we theoretically (through modelling) and experimentally analysed the expected effects of different N forms on plant growth and C and N allocation. We grew *Arabidopsis thaliana* (Arabidopsis) axenically to investigate how root : shoot allocation, root hair formation, and CUE compares between the two N sources NO_3_^−^ and l‐gln. We hypothesized that plants grown on the organic N source would display a reduced N uptake per root mass, increased root biomass and ‐surface area, and an increase in root mass fraction. We used stable isotope labelling (^13^C and ^15^N) to quantify uptake and distribution of N and C sources by plants, enabling assessment of the role of C uptake for the development of an organic N phenotype and enabling calculation of effects of organic N uptake on plant CUE.

## Materials and Methods

### Plant material and growth conditions

In all experiments *A. thaliana* (L.) *Heynh. Col‐0* (wild‐type, WT) plants were used. The seeds were surface sterilized and stratified for 48 h at 4°C. Unless stated otherwise, all plant experiments have been performed using half‐strength N‐free Murashige & Skoog medium (MS) (Murashige & Skoog, 1962), supplemented with 3 mM N in form of either 1.5 mM l‐gln or 3 mM KNO_3_. 1% agar (Duchefa Biochemie, RV Haarlem, the Netherlands) was added after buffering the pH to 5.8 using 7.7 mM 2‐(*N*‐morpholino)ethanesulfonic acid (MES). The medium was free of sucrose. Potassium was compensated for in the l‐gln treatment with addition of KCl equivalent to that in the KNO_3_ treatment.

### Experiment 1: the split‐root experiment

Seeds were germinated on vertical plates filled with half‐strength MS medium supplemented with 3 mM KNO_3_ and 0.5% sucrose. The plants were grown for 14 d under short‐day conditions with an 8 h : 16 h, day : night rhythm (Photosynthetic Photon Flux Density (PPFD) = 200 μmol m^−2^ s^−1^). The primary roots of these 14 d old seedlings were cut to stimulate lateral root development, enabling the establishment of plants in the split‐root system so that similar root biomass would be present in the two root compartments. After seven additional days of growth, the 21 d old seedlings were transferred to the horizontal‐plate, split‐root system and cultivated for additional 14 d.

**Fig. 1:**
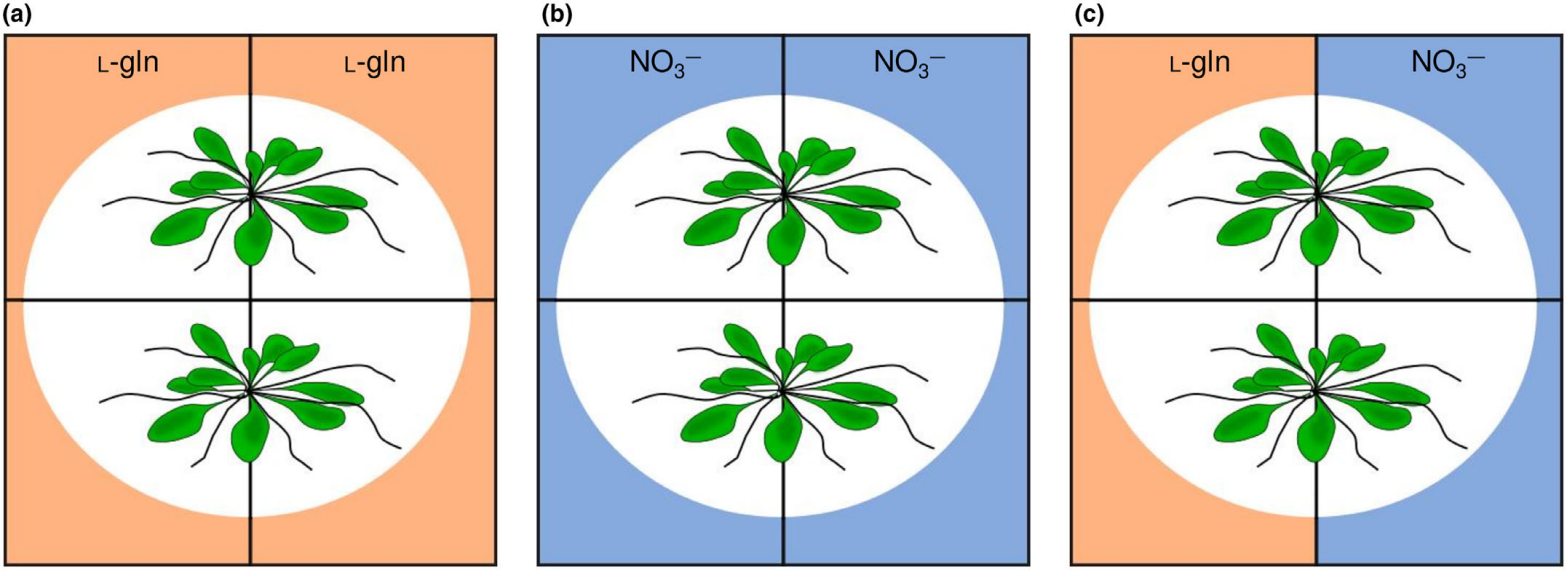
Setup of experiment 1. Shoots were positioned on the middle rib of the plate and roots were divided equally between two growth compartments in which N was supplied as either: (a) 1.5 mM l‐gln on both sides, (b) as 3 mM NO_3_^−^ on both sides or (c) as 1.5 mM l‐gln on one side and 3 mM NO_3_^−^ on the other side. Results shown in Figs 3, 4, 5 and in Table 1 are derived from this experimental system.

For the split‐root system, Petri dishes with two identical separate compartments were filled with half‐strength N‐free MS medium (Fig. 1). Each compartment of the Petri dish was supplemented with 3 mM N in form of 1.5 mM l‐gln or 3 mM KNO_3_ (l‐gln/NO_3_^−^). The medium was free of sucrose. Petri dishes filled exclusively with one N source, 1.5 mM Gln or 3 mM KNO_3_ were used as reference treatments (l‐gln/l‐gln or NO_3_^−^/NO_3_^−^) (Supporting Information Fig. S1). The in total 35 d old plants had root hair length evaluated during the experiment and were then harvested, dried at 60°C and prepared for biomass, N and C concentration.

### Experiment 2

**Fig. 2:**
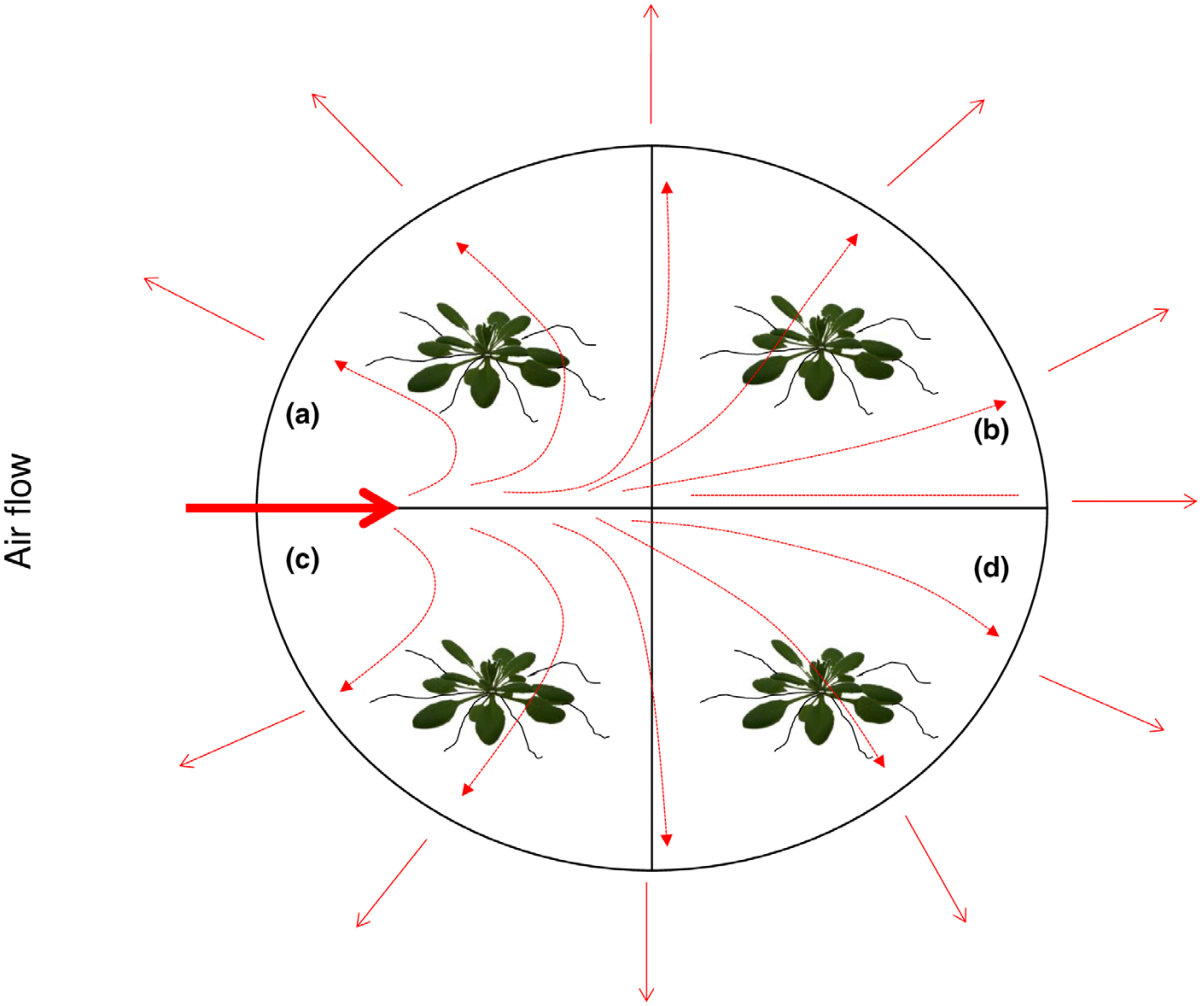
Setup of experiment 2. ^13^C, ^15^N labelling to study uptake of C and N from l‐gln. Seedlings precultivated on vertical plates and moved to plates with air flow. N was supplied as (a) 1.5 mM U^15^N_2_^13^C_5_ – l‐gln (10 atom% enrichment) or on (b) 1.5 mM U^15^N_2_^13^C_5_ – l‐gln + 3 mM NO_3_^−^. Control seedlings grown on (c) nonlabelled l‐gln or on (d) nonlabelled l‐gln + NO_3_^−^, to account for re‐fixation of respired ^13^CO_2_ were included in each plate. Results shown in Figs 6, 7, 8 and Supporting Information Figs S1 and S2 are derived from this experimental system.

Seeds were germinated and plants were grown on horizontal plates containing half‐strength MS medium supplemented with 3 mM KNO_3_ and 0.5% sucrose for 21 d. Then the seedlings were transferred to four section Petri dishes containing either 1.5 mM universally labelled l‐gln (^15^N and ^13^C; 10 atom% excess of each) or a mixture of universally labelled l‐gln (1.5 mM) + KNO_3_ (3 mM) (Fig. 2). A filtered (0.22 μm) air input was connected to each plate in order to avoid respired ^13^CO_2_ to accumulate inside the system. Furthermore, control seedlings grown on nonlabelled l‐gln were also included in each plate to account for re‐fixation of respired ^13^CO_2_. This experiment lasted either 1, 3 or 6 d and consisted of 13 biological replicates for the reference treatments and 26 biological replicates for the l‐gln/NO_3_^−^ treatment, each biological treatment including 60 technical replicates.

### Measurements of N, ^15^N, C and ^13^C

For N and C analyses the samples were ground and homogenized. Analyses were conducted using an Elemental Analyzer – Isotope Ration Mass Spectrometer (EA‐IRMS) (EA: Flash EA 2000, IRMS: DeltaV, both from Thermo Fisher Scientific, Waltham, MA, USA) (Werner *et al*., 1999).

### Root hair length measurement

Root hair development was analysed during the course of the organic and inorganic split‐root experiment. Pictures of roots were taken 7 d after the transfer to the split‐root growth system (28 d old plants), using a Leica DC300 digital camera coupled to a Leica MZ95 stereomicroscope (Leica Microsystems GmbH, Wetzlar, Germany). From each Petri dish compartment, 1–2 pictures of representative areas were taken. Only pictures of 28 d old seedlings were taken due to the high density of roots and root hairs in some treatments in later stages of the experiment. 600–2000 root hairs were measured per treatment using the program ImageJ 1.43 (http://imagej.nih.gov/ij/, Wayne Rasband, National Institute of Mental Health, Bethesda, MD, USA). For this, 18–20 biological replicates have been analysed, each consisting of 60 technical replicates per treatment.

### Calculations and data analysis

Isotopic data (atom% ^13^C and atom% ^15^N) was used to calculate the fraction of plant tissue C and plant tissue N derived from l‐gln, considering the label intensity (10 atom% excess for ^13^C and ^15^N). All data were analysed using the Jmp Pro 16.0.0 software performing a one‐way ANOVA followed by Tukey's *post hoc* test to evaluate the significance. Bars marked with different letters indicate significant differences at *P*‐value ≤ 0.05.

### Estimation of the effects of N form on N assimilation costs and carbon use efficiency

Carbon use efficiency of biomass growth (CUE) is equal to the fraction of C taken up (*C*_u_) that remains in the biomass (*C*_b_), that is CUE = *C*_b_/*C*_u_. CUE was calculated based on the ^13^C : ^15^N ratio in biomass compared to l‐gln molecules (C : N for l‐gln = 0.47 g C g^−1^ N). Assuming all N taken up remains in the biomass, the observed CUE_o_ = ^13^C : ^15^N biomass/^13^C : ^15^N l‐gln.

To quantify the effect of N assimilation carbon costs on CUE, we modelled CUE as a function of the different assimilation costs of C, organic N, and inorganic N (*C*_c_, *oN*_c_, *iN*_c_, respectively) and their contribution to biomass (*C*_b_, *oN*_b_, *iN*_b_, respectively). The total C cost of biomass growth (*C*_ctot_) is the sum *C*_ctot_ = *C*_c_ *C*_b_ + *oN*_c_ *oN*_b_ + *iN*_c_ *iN*_b_. The net C ending up in biomass is *C*_b_ = *C*_u_ − *C*_ctot_, which is combined with the expression CUE = *C*_b_/*C*_u_ to yield the equation for modelled CUE_m_ as a function of assimilation costs:

(Eqn 1) $${\mathrm{CUE}}_{m}=C_{b}/\left(C_{c}\;C_{b}+{\mathrm{oN}}_{c}\;{\mathrm{oN}}_{b}+{\text{iN}}_{c}\;{\text{iN}}_{b}+C_{b}\right)$$

The N assimilation costs were estimated by fitting CUE_m_ to CUE_o_. For this we also need an estimate of the C assimilation cost *C*_c_. The overall cost per plant C assimilation in biomass including associated N assimilation and other processes (the growth respiration), has been estimated to 0.43 (Choudhury, 2001). Because *C*_c_ is the cost excluding N assimilation costs, it must be lower than 0.43 and we assumed that *C*_c_ = 0.2. Smaller or larger *C*_c_ slightly affects the estimated average assimilation costs of the two N forms, but not the relative difference between their assimilation costs, which is our main interest here. To be able to use linear fitting (lm function in R software) we made *oN*_c_ and *iN*_c_ linear coefficients by transforming Eqn 1 to (1/CUE_o_ – *C*_c_ – 1) *C*_b_ = *oN*_c_ *oN*_b_ + *iN*_c_ *iN*_b_.

## Results

### Organic N causes changes in plant phenotype

To test whether plant available organic or inorganic N forms affect a plant's phenotype differently, *A. thaliana* was grown in a split‐root system, where the roots had access to either only l‐gln (l‐gln/l‐gln), only NO_3_^−^ (NO_3_^−^/NO_3_^−^) or both (l‐gln/NO_3_^−^) (Fig. 1).

**Fig. 3:**
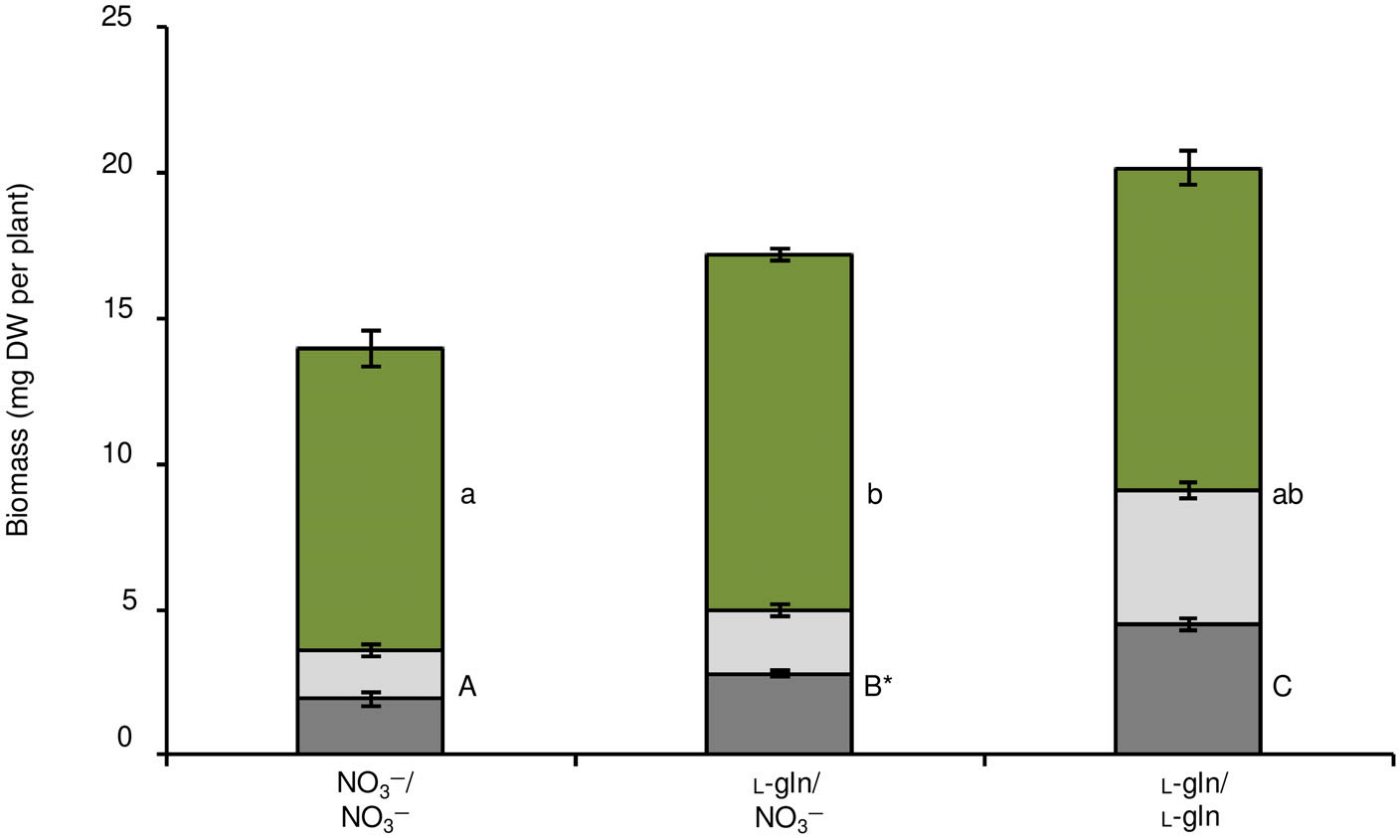
Shoot and root biomass of *Arabidopsis thaliana* plants grown on axenic split‐root systems. Roots were divided equally between two growth compartments containing agar media with N administered either as 3 mM nitrate in both root compartments (NO_3_^−^/NO_3_^−^), *n* = 5, as 3 mM nitrate in one of the root compartments and 1.5 mM l‐gln in the other compartment (l‐gln/NO_3_^−^), *n* = 10, or as 1.5 mM l‐gln (l‐gln/l‐gln) in both root compartments, *n* = 5. Green, upper part of the bars correspond to shoots, light grey, middle part of the bars to l‐gln root compartment in the NO_3_^−^/l‐gln treatment and lower grey part of the bars correspond to roots in the NO_3_^−^ compartment in the l‐gln/NO_3_^−^ treatment. Bars represent average ± SE. Statistical significance was calculated using one‐way ANOVA and Tukey *post hoc* test. Different lower‐case and upper‐case letters indicate significant differences at *P*‐value ≤ 0.05 in shoot and root biomass between treatments, respectively. The * indicates a statistical difference in root biomass between root compartments in the l‐gln/NO_3_^−^ treatment. DW, dry weight.

The root biomass of plants differed significantly between the N sources (Fig. 3). Plants grown solely on organic N (l‐gln/l‐gln) demonstrated a higher root biomass compared to plants with access to NO_3_^−^. However, seedlings with access to both N sources (l‐gln/NO_3_^−^) showed opposing responses, the root side with access to NO_3_^−^ had significantly higher biomass compared to the root side exposed to l‐gln. In addition to that, plants grown solely on l‐gln did not significantly differ in shoot biomass between the different N treatments. However, plants grown on both N sources (l‐gln/NO_3_^−^) displayed significantly higher shoot biomass compared to plants grown solely on NO_3_^−^ (Fig. 3). The shoot biomass production was influenced by the availability of l‐gln and also the total biomass was enhanced by the availability of l‐gln.

**Fig. 4:**
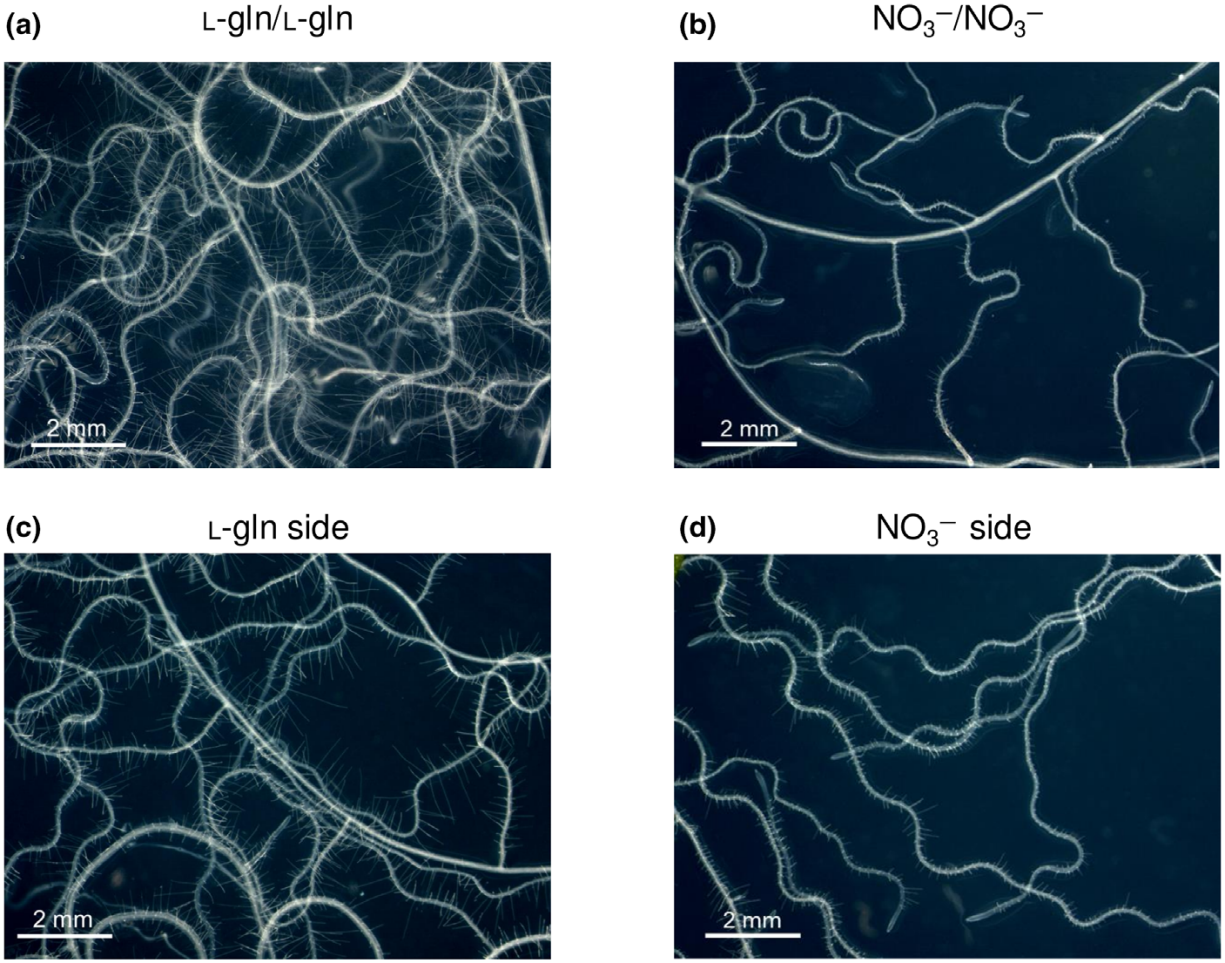
Root systems of *Arabidopsis thaliana* plants grown in split‐root systems with N supplied as (a) 1.5 mM l‐gln supplied on both sides (l‐gln/l‐gln), (b) 3 mM NO_3_^−^ on both sides (NO_3_^−^/NO_3_^−^), as or as N supplied as (c) 1.5 mM l‐gln on one side and supplied (d) 3 mM NO_3_^−^ on the other side. Pictures were taken using a Leica DC300 digital camera coupled to a Leica MZ9_5_ stereomicroscope.

**Fig. 5:**
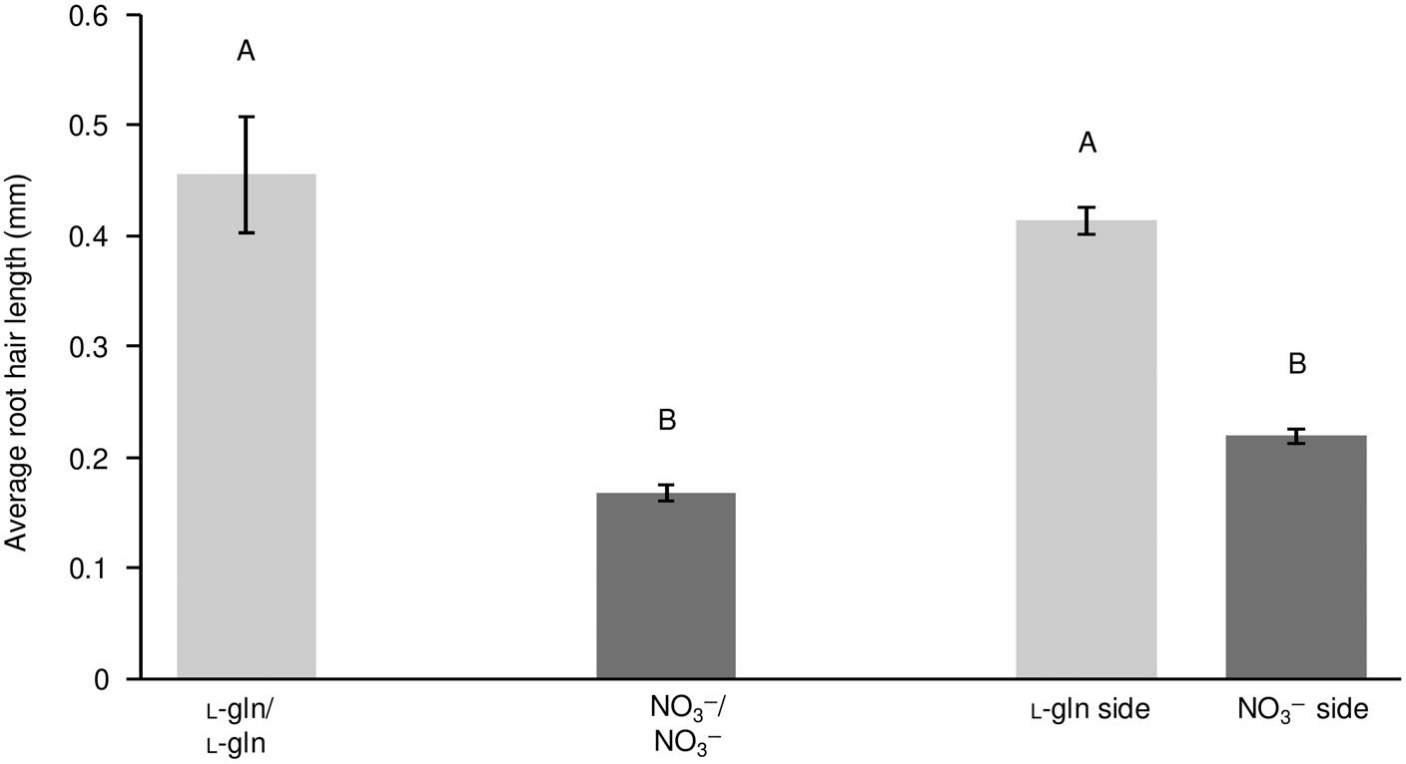
Root hair length of *Arabidopsis thaliana* plants with N supplied as 1.5 mM l‐gln supplied on both sides (l‐gln/l‐gln), 3 mM NO_3_^−^ on both sides (NO_3_^−^/NO_3_^−^), as or as N supplied as 3 mM NO_3_^−^ on one side and 1.5 mM l‐gln supplied on the other side. Pictures of roots from 18 to 20 plants of each treatment were analysed and root hair length was measured using the program ImageJ. Values indicate average ± SE (*n* = 18–20). Statistical significance was calculated using one‐way ANOVA and Tukey *post hoc* test. Different capital letters indicate statistical differences at *P*‐value ≤ 0.05 in root hair length between N treatments.

Plants grown exclusively on l‐gln, developed elongated root hairs compared to plants that only had access to inorganic N (Fig. 4a,b). Plant roots with access to both N sources (l‐gln/NO_3_^−^) exhibited similar responses as roots that had access to a single N source only (Fig. 4c,d). However, the root development was not affected by the corresponding N source on the other root side. Root hair length measurements confirmed these observations (Fig. 5). Seedlings grown solely on l‐gln (l‐gln/l‐gln) displayed significantly increased root hair length compared to seedlings grown on NO_3_^−^ as the sole N source (l‐gln/l‐gln = 0.45 ± 0.05 mm, NO_3_^−^/NO_3_^−^ = 0.17 ± 0.01 mm; Fig. 5). A positive effect of l‐gln on root hair length was also visible for plants having access to both N sources (l‐gln/NO_3_^−^): The root side with access to organic N had significantly longer root hairs compared to the side which had access to NO_3_^−^ (l‐gln side = 0.41 ± 0.01 mm, NO_3_^−^ side = 0.22 ± 0.01 mm). These results demonstrated that plants develop a unique root phenotype with increased root hair length when exposed to organic N (Fig. 4).

### Organic N plants display similar N status but higher C concentration

Analysis of N and C contents of plants in the split‐root experiment revealed that those grown on l‐gln (l‐gln/l‐gln), or mixtures of l‐gln and NO_3_^−^ (l‐gln/NO_3_^−^) had shoot‐N concentrations equally high as those grown on NO_3_^−^ only (Table 1). However, root N concentrations were lower for plants grown on l‐gln and in the mixed N treatment, roots supplied l‐gln displayed lower N concentrations than those supplied NO_3_^−^ (Table 1). Carbon concentrations followed the opposite pattern, being higher for all treatments and both organs for plants supplied l‐gln. Interestingly, in the mixed N treatment, roots supplied NO_3_^−^ also exhibited increased C concentrations (Table 1).

**Table 1:** Nitrogen and carbon concentrations of shoots and roots of *Arabidopsis thaliana* plants grown in split‐root systems.

Treatment	Organ	N concentration (% DW)	C concentration (% DW)	No. of replicates
NO_3_^−^/NO_3_^−^	Shoot	6.37 (0.09) A	35.01 (0.74) A	5
	Root	4.54 (0.05) a	38.14 (0.21) a	5
l‐gln/l‐gln	Shoot	6.62 (0.13) A	39.23 (0.33) B	5
	Root	3.14 (0.11) b	40.24 (0.21) b	5
l‐gln/NO_3_^−^	Shoot	6.54 (0.07) A	35.61 (0.49) A	10
	Root l‐gln side	3.24 (0.10) b	39.99 (0.36) b	10
	Root NO_3_^−^ side	4.15 (0.24) a	39.92 (0.31) b	10

Roots were divided equally between two growth compartments containing agar media. Nitrogen (3 mM) was supplied to roots either exclusively as nitrate (NO_3_^−^/NO_3_^−^ treatment) or exclusively as glutamine (l‐gln/l‐gln treatment) or as nitrate in one of the root compartments and on the other (l‐gln/NO_3_^−^ treatment). Values represent mean ± SE, *n* = 5–10. Different letters indicate significant differences (*P*‐value ≤ 0.05) between treatments for shoots (upper‐case letters) and roots (lower‐case letters). DW, dry weight.

### Stable isotope labelling shows that organic N contributes to plant C

**Fig. 6:**
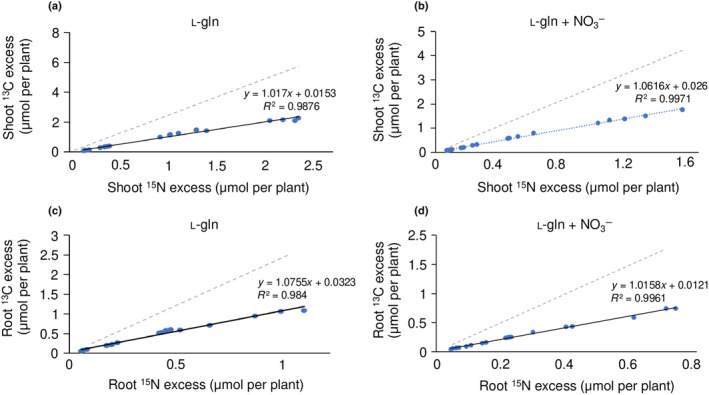
Regression analysis of excess ^13^C vs excess ^15^N content in *Arabidopsis thaliana* plants grown on 1.5 mM U^15^N_2_U^13^C_5_‐l‐gln (10 atom%; a, c) or a mixture of 0.75 mM, U^15^N_2_U^13^C_5_‐l‐gln (10 atom%) and 1.5 mM nitrate (nonlabelled; b, d). Dotted lines with slope 2.5 indicate theoretical regressions corresponding to all ^13^C acquired through uptake of l‐gln remaining in tissues. Regression equations (a) *y* = 1.017*x* + 0.015 (*R* ^2^ = 0.99); (b) *y* = 1.062*x* + 0.03 (*R* ^2^ = 1.0); (c) *y* = 1.075*x* + 0.03 (*R* ^2^ = 0.99); (d) 1.016 + 0.012 (*R* ^2^ = 1.0). Slopes correspond to (a) 41%; (b) 42%; (c) 43% and (d) 41% of carbon derived from uptake of l‐gln remaining in plant biomass.

We traced the uptake and partitioning of C and N from organic N, growing Arabidopsis on ^13^C, ^15^N labelled l‐gln (U^15^N_2_U^13^C_5_‐l‐gln) either as the sole N source (Fig. 6a,c) or in a 50 : 50 (moles of N) mixture with NO_3_^−^ (Figs 2, 6b,d). The ^15^N abundance in the growth medium was 10 atom% and in agreement with this, the slope of the regression line total plant N vs excess ^15^N for shoots and roots of plants growing on labelled l‐gln was 0.098 and 0.102 (Fig. S1a,b, respectively). The corresponding slope for shoots and roots of plants growing on an equimolar N mixture of labelled l‐gln and NO_3_^−^ was 0.06 and 0.08 respectively, that is higher than 0.05 which would correspond to identical uptake rates of NO_3_^−^ and l‐gln (Fig. S2a,b, respectively), suggesting a higher rate of acquisition of l‐gln than of NO_3_^−^.

Regression analysis of excess ^13^C vs excess ^15^N content in plants grown on 1.5 mM U^15^N_2_U^13^C_5_‐l‐gln or a mixture of 0.75 mM, U^15^N_2_U^13^C_5_‐l‐gln (10 atom%) and 1.5 mM NO_3_^−^ showed that between 41% and 43% of the C acquired from uptake of l‐gln remained in the tissues (Fig. 6).

### The costs and benefits of organic vs inorganic N: N assimilation costs, carbon use efficiency, and N uptake

**Fig. 7:**
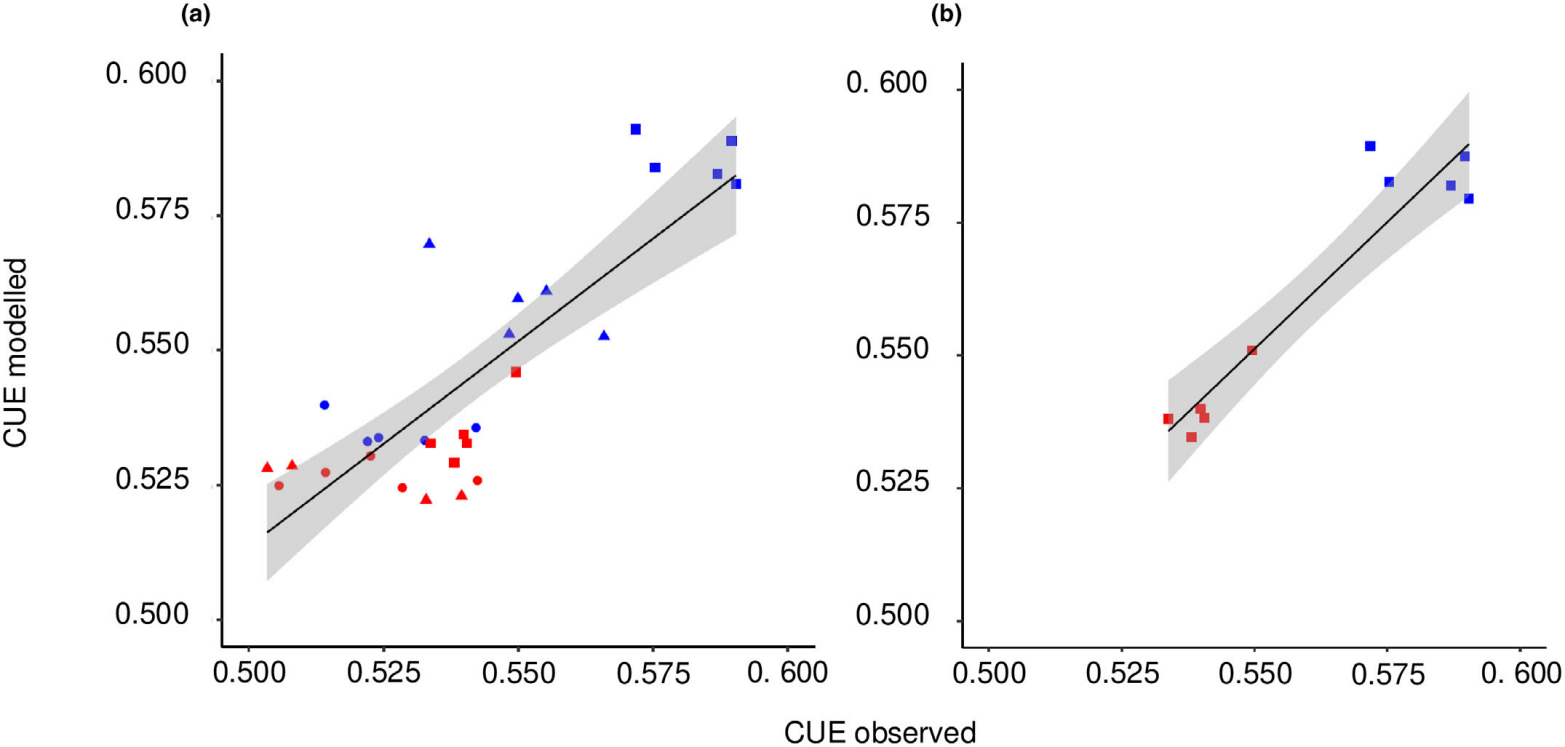
Carbon use efficiency (CUE) modelled based on N assimilation costs vs observed CUE for *Arabidopsis thaliana* plants grown on 1.5 mM l‐gln (blue symbols) or mixed l‐gln and NO_3_^−^ (0.75 and 1.5 mM, respectively; red symbols). The growing times were 1 d (circles), 3 d (triangles) and 6 d (squares). (a) All observations, *R* ^2^ = 0.66, (b) only observations at day 6, *R* ^2^ = 0.89. The shaded areas indicate a 95% confidence band of the mean.

**Fig. 8:**
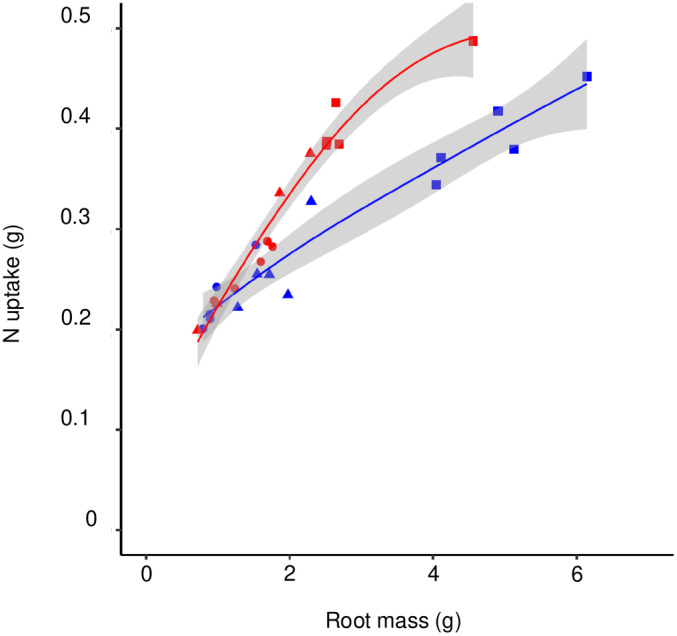
N uptake vs root mass for *Arabidopsis thaliana* plants grown on 1.5 mM l‐gln (blue symbols) or mixed l‐gln and NO_3_^−^ (0.75 and 1.5 mM, respectively; red symbols), for 1 d (circles), 3 d (triangles) and 6 d (squares). The shaded area indicates a 95% confidence band of the mean.

Carbon use efficiency of biomass growth increased with the fraction of N that was taken up as l‐gln relative to NO_3_^−^ and the difference was mainly explained by the difference in N assimilation costs between N forms (Fig. 7). As the influence of pre‐experimental differences between plants declined over time, the correlation between CUE and N form increased. After 6 d of growth, the lower N assimilation cost of l‐gln compared to NO_3_^−^ explained as much as 89% of the difference in CUE. The estimated N assimilation costs were 2.63 ± 0.10 g C g^−1^ N for l‐gln and 4.56 ± 0.25 g C g^−1^ N for NO_3_^−^. N taken up at a given root biomass was *c*. 20% higher for plants growing on the mixed N than for those growing on l‐gln only (Fig. 8).

## Discussion

Changes in root : shoot ratios and root morphology in response to N supply is a well‐documented phenomenon (Hermans *et al*., 2006; Lynch *et al*., 2023). The general view is that plants adjust biomass allocation between above‐ and belowground structures to optimize acquisition of the limiting resource; the functional balance or functional equilibrium hypothesis (Brouwer, 1962; Poorter *et al*., 2012). Root phenotypic responses to N supply have been extensively studied in Arabidopsis, but most of these studies have concerned mineral N and in particular NO_3_^−^. From these reports, it was concluded that increasing N supply leads to a reduction in the root mass fraction, reduced lateral root formation and fewer active root tip meristems (Jia & von Wirén, 2020). Through the series of experiments described here, we challenge the view that N supply rates is the single determinant of root morphology and architecture and propose that the source of N (inorganic or organic) available to plants exerts a strong influence on plant phenotype.

Growing Arabidopsis from seeds on vertical plates and then transferring them to horizontal plates, with split‐root setups (Fig. 1), revealed that growth of roots was significantly enhanced by l‐gln, leading to a higher total plant biomass (Fig. 3). Our results also show that a key facet of root morphology; root hair length, responds strongly to the supply of l‐gln. While the abundance of root hairs was similar between roots exposed to l‐gln and NO_3_^−^, root hairs were nearly three times longer for plants grown on l‐gln vs plants grown on NO_3_^−^ and *c*. 2 times longer for roots in the split‐root setup supplied l‐gln vs roots supplied NO_3_^−^ (Figs 4, 5). This corresponds to a significant increase in root surface area, a characteristic that would also have a strong fitness value for growth on organic N and for survival under drought (Choi & Cho, 2019). Our estimate of root hair length for NO_3_^−^‐treated roots is similar to those reported for low‐ and intermediate NO_3_^−^ concentration treated Arabidopsis Col‐0 reported by De Pessemier *et al*. (2022). The average root hair length of roots exposed to l‐gln in our study was twice that reported by these authors, illustrating the strength of the phenotypic response to the organic N source. A recent study reported on various aspects of l‐gln nutrition of Arabidopsis: enhanced stress responses and disease resistance but also a significant increase in lateral root density compared with plants grown on NO_3_^−^ (Lia *et al*., 2024). While there are differences between our experimental system and that of Lia *et al*. in that they used higher l‐gln concentrations (5 mM vs 1.5 mM) and grew plants on vertical plates rather than horizontal plates and for a shorter period (12 d vs 35 d), the notion of an increase in lateral root initiation is in line with our main hypothesis; that l‐gln, as a source of organic N, promotes root proliferation (Fig. 3). The importance of expansion of root surface area for uptake of immobile nutrients has been verified using root hair mutants with short (Gahoonia *et al*., 2001) and long (Zhang *et al*., 2018) root hairs. Future studies may hence use a similar approach to test the role of root hairs for acquisition of immobile organic N sources. Here, we note that the phenotypic response of Arabidopsis to an organic N source is like the well‐documented response to immobile phosphorus and potassium (Gahoonia *et al*., 1997; Gahoonia & Nielsen, 1998; Bates & Lynch, 2001; Jungk, 2001; Bienert *et al*., 2021).

A key question is to what extent the root morphological response to l‐gln documented in our study is calid also for (1) other organic N sources and (2) for other plants, in particular mycorrhizal plants. A study using Arabidopsis and *Hakea actites* (*Proteaceae* nonmycorrhizal) reported increased root length when plants were grown in axenic culture and supplied with a complex organic N source (the protein Bovine Serum Albumin; BSA; Paungfoo‐Lonhienne *et al*., 2008). Increased root hair length in response to presence of BSA in the root medium was reported for Arabidopsis (Lonhienne *et al*., 2014). Thus, at the two ends of the complexity spectra of organic N (single amino acid; l‐gln; the current study and large protein 583 amino acids) plants react with increased root surface area through increased root length and increased length of root hairs. Regarding the generality of response, in particular mycorrhizal plants, a study by Gruffman *et al*. (2012) reported increased root mass fraction as well as increased frequency of mycorrhizal root tips for conifer seedlings (*Pinus sylvestris* and *Picea abies*) when these were cultivated with the amino acid l‐arg as a nitrogen source.

Following absorption of organic N, endogenous metabolism will lead to that a fraction of the acquired C is lost via respiration while the rest is incorporated into biomass. The instantaneous metabolism of l‐gln was shown to produce l‐glu, l‐asp and GABA, leading to a 15% loss of C acquired from l‐gln over a time course of 120 min (Svennerstam & Jämtgård, 2022). Here, we show that between 57 and 59% of the C acquired from uptake of l‐gln was lost through respiration, independently of tissue and independently if N was administered as l‐gln only or as a mixture of N compounds (Fig. 6). The concentration of tissue C derived from l‐gln uptake can be estimated as the product of tissue N concentration × slope of the regression excess ^13^C vs excess ^15^N (Fig. 6). For shoots and roots of plants grown on l‐gln this amounts to 6.7% and 3.4% of DW respectively. This means that 17.2% and 8.4% of shoot and root C was derived from uptake of l‐gln.

A long‐standing debate within the field of N nutrition is whether inorganic N, in particular NO_3_^−^, is the preferred N source for plants (Harrison *et al*., 2007) and that organic N would be of importance only at low‐inorganic N availabilities. The results from our experiment (Figs S1, S2), with N available as mixtures of inorganic and organic N, the opposite result was achieved. Thus, slopes of regression lines for shoots and roots of plants growing on an equimolar N mixture of labelled l‐gln and NO_3_^−^ was 0.06 and 0.08 respectively (Fig. S2a,b, respectively). Re‐calculated, this equals the fraction of N derived from l‐gln was 60% for shoots and 80% for roots l‐gln than of NO_3_^−^. This shows that l‐gln was the preferred N source under the growth conditions used in the experiment. Also, our data illustrates that roots, to a higher extent than shoots, used N absorbed as l‐gln for growth.

A fundamental difference between inorganic and organic N is the C savings and C bonus connected to plant use of organic N. Franklin *et al*. (2017) developed a model based on the differences in C costs for different N sources and suggested this extra C to drive a shift towards an increased root mass fraction. In the current study, the contribution of l‐gln‐derived C to total organ C was assessed on plant grown on U^15^N_2_U^13^C_5_‐l‐gln. The ratio of the two isotopes ^13^C to ^15^N in l‐gln, and hence in the growth media was 2.5. This means that if absorbed l‐gln was not metabolized by the plant following uptake, we would expect the ratio of excess ^13^C and excess ^15^N to equal 2.5 in plants and that any deviation from 2.5 would be due to losses of ^13^C via catabolism of l‐gln. The slopes of regressions of excess ^13^C vs excess ^15^N provides information here, and results showed similar slopes for both roots and shoots and for both plants grown on l‐gln and plants grown on a combination of l‐gln and NO_3_^−^ (Fig. 6). Re‐calculated, these slopes (1.02–1.08; Fig. 6) correspond to 40.8–43.2 of the C acquired as l‐gln remaining in tissues. The ^13^C data was also used to calculate the fraction of plant C that was derived from l‐gln uptake. For plants grown on l‐gln, as the sole N source, *c*. 10% of root C was derived from l‐gln at the final harvest (10 d after plants had been moved to the labelled source). These results show that uptake of l‐gln made a significant contribution to plant C and to root C.

Carbon use efficiency is a key metric describing the efficiency by which photosynthetically derived C is converted to biomass C (Manzoni *et al*., 2018). For small plants, assimilation of inorganic N, in particular NO_3_^−^, may constitute a significant C cost (Bloom *et al*., 2003) and this would hence also potentially affect plant CUE. The carbon bonus of organic N nutrition, as described by Franklin *et al*. (2017) and further demonstrated here, pertains both to the C acquired through uptake of organic N but also to the C savings derived from not having to reduce NO_3_^−^ to NO_2_^−^ via nitrate reductase and further to NH_4_^+^ via nitrite reductase as well as the assimilation of NH_4_^+^ via the glutamine synthetase (GS)/glutamate synthase (GOGAT) pathway. In line with this reasoning, we show here that CUE of small Arabidopsis plants was positively correlated with the degree of l‐gln assimilation (Fig. 7). Moreover, measured CUE was well predicted based on differences in C costs for assimilation between l‐gln and NO_3_^−^, explaining 89% of the difference after 6 d of growth (Fig. 7). In addition, the validity of the estimated N assimilation costs was supported by their similarity to estimates based on the underlying biochemical reactions, that is 2.16 and 5.81 (Zerihun *et al*., 1998). These results indicate that N assimilation costs are decisive for CUE of small plants and are clearly affected by the differences in assimilation costs of different N sources.

While substantial C savings may come from uptake of organic N, their lower mobility also incurs C costs. Acquisition of N is chiefly through mass flow and diffusion, the former primarily of importance for N forms that occur in substantial amounts in the soil solution, mainly NO_3_^−^. Our data suggest root N uptake per unit root mass to be higher for NO_3_^−^ than for l‐gln (Fig. 8), implying a higher C cost per N uptake for organic than inorganic N, which is only partly alleviated by the longer root hairs. This effect would be further aggravated under conditions allowing for mass flow (McMurtrie & Näsholm, 2018), which would not occur in the test system used here but which would limit the benefits of longer root hairs.

We conclude that strong differences in soil mobility may have exerted a selection pressure for plant plasticity in root allocation and root architecture not only linked to the availability of N but also to the chemical composition of available N. This plasticity is manifested through increases in root mass fraction, root branching and extension of root surface area through root hairs, enabling enhanced uptake of compounds of lower mobility. At the same time, these exact responses result in lower rates of mass flow‐mediated N gain, suggesting plants face a trade‐off between acquisition of less mobile and mobile N sources.

The demand for higher root surface areas to optimize uptake of organic N incurs an additional C cost for plants, potentially reducing growth rates. However, the substantially lower cost for N assimilation of organic N, leading to a higher CUE, alleviates this negative effect.

Overall, our results show that in a whole plant perspective, the two key differences between organic and inorganic N, (1) a lower N uptake per unit root biomass and (2), a lower N assimilation cost leading to higher CUE, make a higher root mass fraction an inevitable consequence for the plant to maintain a balanced N : C ratio during growth.

## Competing interests

TN declares a competing interest as he owns shares in, and works part time for, the company Arevo AB that develops, produces, and markets organic fertilizers. RG also declares a competing interest as she is also employed by Arevo AB. All other authors declare that the research was performed without any conflicting commercial or financial relationships and hence declare no conflict of interest.

## Author contributions

LT, CAC, TN and RG designed the project. LT, CAC, PM and RG performed the experiments. LT, CAC, OF, PM, TN and RG analysed the data. LT, CAC and TN wrote the initial draft with input from all other authors. All co‐authors provided feedback and revised the manuscript. LT and CAC contributed equally to this work.

## Supporting information


**Fig. S1** Regression analysis of excess ^15^N vs total N contents of 10 atom% excess U^15^N_2_U^13^C_5_‐l‐gln grown *Arabidopsis thaliana* plants.
**Fig. S2** Regression analysis of excess ^15^N vs total N contents of *Arabidopsis thaliana* plants grown on a mixture of 0.75 mM 10 atom% excess U^15^N_2_U^13^C_5_‐l‐gln and 1.5 mM nonlabelled NO_3_^−^.
Please note: Wiley is not responsible for the content or functionality of any Supporting Information supplied by the authors. Any queries (other than missing material) should be directed to the *New Phytologist* Central Office.

## Data Availability

All data used in this study are uploaded to a data repository and can be accessed at doi: 10.5281/zenodo.13740411.

## References

[nph20285-bib-0001] Ågren GI , Ingestad T . 1987. Root:shoot ratio as a balance between nitrogen productivity and photosynthesis. Plant, Cell & Environment 10: 579–586.

[nph20285-bib-0002] Bates TR , Lynch JP . 2001. Root hairs confer a competitive advantage under low phosphorus availability. Plant and Soil 236: 243–250.

[nph20285-bib-0003] Bhat KKS , Nye PH , Bereton AJ . 1979. The possibility of predicting solute uptake and plant growth responses from independently measured soil and plant characteristics. VI. The growth and uptake of rape in solution of constant nitrate concentration. Plant and Soil 53: 137–167.

[nph20285-bib-0004] Bienert MD , Werner KM , Wimmer MA , Bienert GP . 2021. Root hairs: the villi of plants. Biochemical Society Transactions 49: 1133–1146. 34013353 10.1042/BST20200716

[nph20285-bib-0005] Bloom AJ , Meyerhoff PA , Taylor AR , Rost TL . 2003. Root development and the absorption of ammonium and nitrate from the rhizosphere. Journal of Plant Growth Regulation 21: 416–431.

[nph20285-bib-0006] Brouwer R . 1962. Nutritive influences on the distribution of dry matter in the plant. Netherlands Journal of Agricultural Science 10: 399–408.

[nph20285-bib-0007] Choi HS , Cho HT . 2019. Root hairs enhance Arabidopsis seedling survival upon soil disruption. Scientific Reports 9: 11181. 31371805 10.1038/s41598-019-47733-0 PMC6671945

[nph20285-bib-0008] Choudhury BJ . 2001. Implementing a nitrogen‐based model for autotrophic respiration using satellite and field observations. Tropical Ecology 2: 141–174.

[nph20285-bib-0009] De Pessemier J , Moturu TR , Nacry P , Ebert R , De Gernier H , Tillard P , Swarup K , Wells DM , Haseloff J , Murray SC *et al*. 2022. Root system size and root hair length are key phenes for nitrate acquisition and biomass production across natural variation in Arabidopsis. Journal of Experimental Botany 73: 3569–3583. 35304891 10.1093/jxb/erac118

[nph20285-bib-0010] Ewens M , Leigh RA . 1985. The effect of nutrient solution composition on the length of root hairs of wheat (*Triticum aestivum* L.). Journal of Experimental Botany 36: 713–724.

[nph20285-bib-0011] Foehse D , Jungk A . 1983. Influence of phosphate and nitrate supply on root hair formation of rape, spinach and tomato plants. Plant and Soil 74: 359–368.

[nph20285-bib-0012] Franklin O , Cambui C , Palmroth S , Oren R , Näsholm T . 2017. The carbon bonus of organic nitrogen enhances nitrogen use efficiency of plants. Plant, Cell & Environment 40: 25–35. 10.1111/pce.12772 PMC5217072 27241731

[nph20285-bib-0013] Gahoonia TS , Care D , Nielsen NE . 1997. Root hairs and phosphorus acquisition of wheat and barley cultivars. Plant and Soil 191: 181–188.

[nph20285-bib-0014] Gahoonia TS , Nielsen NE . 1998. Direct evidence on participation of root hairs in phosphorus (^32^P) uptake from soil. Plant and Soil 198: 147–152.

[nph20285-bib-0015] Gahoonia TS , Nielsen NE , Joshi PA , Jahor A . 2001. A root hairless barley mutant for elucidating genetic control of root hairs and phosphorus uptake. Plant and Soil 235: 211–219.

[nph20285-bib-0016] Gruffman L , Nordin A , Ishida T , Näsholm T . 2012. Cultivation of Norway spruce and Scots pine on organic nitrogen improves seedling morphology and field performance. Forest Ecology & Management 276: 118–124.

[nph20285-bib-0017] Harrison KA , Bol R , Bardgett RD . 2007. Preferences for different nitrogen forms by coexisting plant species and soil microbes. Ecology 88: 989–999. 17536714 10.1890/06-1018

[nph20285-bib-0018] Hermans C , Hammond JP , White PJ , Verbruggen N . 2006. How do plants respond to nutrient shortage by biomass allocation? Trends in Plant Science 11: 610–617. 17092760 10.1016/j.tplants.2006.10.007

[nph20285-bib-0019] Jia Z , von Wirén N . 2020. Signaling pathways underlying nitrogen‐dependent changes in root system architecture: from model to crop species. Journal of Experimental Botany 2020: 4393–4404. 10.1093/jxb/eraa033 PMC7382383 31970412

[nph20285-bib-0020] Jungk A . 2001. Root hairs and the acquisition of plant nutrients from soil. Journal of Plant Nutrition and Soil Science 164: 121–129.

[nph20285-bib-0021] Kiba T , Krapp A . 2016. Plant nitrogen acquisition under low availability: regulation of uptake and root architecture. Plant & Cell Physiology 57: 707–714. 27025887 10.1093/pcp/pcw052 PMC4836452

[nph20285-bib-0022] Lia H‐S , Lee K‐T , Chung Y‐H , Chen S‐Z , Hung Y‐J , Hsieh M‐H . 2024. Glutamine induces lateral root initiation, stress responses, and disease resistance in Arabidopsis. Plant Physiology 195: kiae144. 10.1093/plphys/kiae144 38466723

[nph20285-bib-0023] Lonhienne TGA , Trusov Y , Young A , Rentsch D , Näsholm T , Schmidt S , Paungfoo‐Lonhienne C . 2014. The effect of protein on root morphology and plant biomass allocation. Scientific Reports 4: 5055. 24852366 10.1038/srep05055 PMC4031471

[nph20285-bib-0024] Lynch JP , Galindo‐Castañeda T , Schneider HM , Sidhu JS , Rangarajan H , York LM . 2023. Root phenotypes for improved nitrogen capture. Plant and Soil 502: 31–85. 39323575 10.1007/s11104-023-06301-2 PMC11420291

[nph20285-bib-0025] Manzoni S , Čapek P , Porada P , Thurner M , Winterdahl M , Beer C , Brüchert V , Frouz J , Herrmann AM , Lindahl BD *et al*. 2018. Reviews and syntheses: carbon use efficiency from organisms to ecosystems – definitions, theories, and empirical evidence. Biogeosciences 15: 5929–5949.

[nph20285-bib-0026] McMurtrie RE , Näsholm T . 2018. Quantifying the contribution of mass flow to nitrogen acquisition by an individual plant root. New Phytologist 218: 119–130. 29226964 10.1111/nph.14927

[nph20285-bib-0027] Murashige T , Skoog F . 1962. A revised medium for rapid growth and bio assays with tobacco tissue cultures. Physiologia Plantarum 15: 473–497.

[nph20285-bib-0028] Nye PH . 1977. The rate‐limiting step in plant nutrient absorption from soil. Soil Science 123: 292–297.

[nph20285-bib-0029] Paungfoo‐Lonhienne C , Lonhienne TGA , Rentsch D , Robinson N , Christie M , Webb RI , Gamage HK , Carroll BJ , Schenk PM , Schmidt S . 2008. Plants can use protein as a nitrogen source without assistance fromother organisms. Proceedings of the National Academy of Sciences, USA 105: 4524–4529. 10.1073/pnas.0712078105 PMC2393761 18334638

[nph20285-bib-0030] Poorter H , Niklas KJ , Reich PB , Oleksyn J , Poot P , Mommer L . 2012. Biomass allocation to leaves, stems and roots: meta‐analyses of interspecific variation and environmental control. New Phytologist 193: 30–50. 22085245 10.1111/j.1469-8137.2011.03952.x

[nph20285-bib-0031] Reynolds HL , D'Antonio C . 1996. The ecological significance of plasticity in root weight ratio in response to nitrogen: opinion. Plant and Soil 185: 75–97.

[nph20285-bib-0032] Saengwilai P , Strock C , Rangarajan H , Chimungu J , Salungyu J , Lynch JP . 2021. Root hair phenotypes influence nitrogen acquisition in maize. Annals of Botany 128: 849–858. 34355736 10.1093/aob/mcab104 PMC8577201

[nph20285-bib-0033] Smith S , De Smet I . 2012. Root system architecture: insights from Arabidopsis and cereal crops. Philosophical Transactions of the Royal Society of London. Series B: Biological Sciences 367: 1441–1452. 22527386 10.1098/rstb.2011.0234 PMC3321685

[nph20285-bib-0034] Svennerstam H , Jämtgård S . 2022. Timing is everything – obtaining accurate measures of plant uptake of amino acids. New Phytologist 234: 311–318. 35023179 10.1111/nph.17964 PMC9303729

[nph20285-bib-0035] Tinker PB , Nye PH . 2000. Solute movement in the rhizosphere. New York, NY, USA: Oxford University Press.

[nph20285-bib-0036] Vicca S , Luyssaert S , Peñuelas J , Campioli M , Chapin FS III , Ciais P , Heinemeyer A , Högberg P , Kutsch WL , Law BE *et al*. 2012. Fertile forests produce biomass more efficiently. Ecology Letters 15: 520–526. 22472207 10.1111/j.1461-0248.2012.01775.x

[nph20285-bib-0037] Werner RA , Bruch BA , Brand WA . 1999. ConFlo III – an interface for high precision δ^13^C and δ^15^N analysis with an extended dynamic range. Rapid Communications in Mass Spectrometry 13: 1237–1241. 10407304 10.1002/(SICI)1097-0231(19990715)13:13<1237::AID-RCM633>3.0.CO;2-C

[nph20285-bib-0038] Zerihun A , McKenzie BA , Morton JD . 1998. Photosynthate costs associated with the utilization of different nitrogen‐forms: influence on the carbon balance of plants and shoot‐root biomass partitioning. New Phytologist 138: 1–11.

[nph20285-bib-0039] Zhang C , Simposon RJ , Kim CM , Warthmann N , Delhaize E , Dolan L , Byrne ME , Wu Y , Ryan PR . 2018. Do longer root hairs improve phosphorus uptake? Testing the hypothesis with transgenic *Brachypodium distachyon* lines overexpressing endogenous RSL genes. New Phytologist 217: 1654–1666. 29341123 10.1111/nph.14980

