# Supplementary material for: Plant organic nitrogen nutrition: costs, benefits, and carbon use efficiency
Source: New Phytol. 2024 Nov 15;245(3):1018–28. doi: 10.1111/nph.20285 (PMC11711965; doi:10.1111/nph.20285)
Supplement: Supplementary file 1 — Fig. S1 Regression analysis of excess 15N vs total N contents of 10 atom% excess U15N2U13C5‐l‐gln grown Arabidopsis thaliana plants. Fig. S2 Regression analysis of excess 15N vs total N contents of Arabidopsis thaliana plants grown on a mixture of 0.75 mM 10 atom% excess U15N2U13C5‐l‐gln and 1.5 mM nonlabelled NO3 −. Please note: Wiley is not responsible for the content or functionality of any Supporting Information supplied by the authors. Any queries (other than missing material) should be directed to the New Phytologist Central Office. [file NPH-245-1018-s001.docx]

## *New Phytologist* Supporting Information

Article title: Plant organic nitrogen nutrition: costs, benefits, and carbon use efficiency

Authors: Laura Tünnermann, Camila Aguetoni Cambui, Oskar Franklin, Patrizia Merkel, Torgny Näsholm, Regina Gratz

Article acceptance date: 30 October 2024

The following Supporting Information is available for this article:

**Fig. S1** Regression analysis of excess ^15^N vs. total N contents of 10 atom% excess U^15^N_2_U^13^C_5_-L-gln grown *Arabidopsis thaliana* plants.

**Fig. S2** Regression analysis of excess ^15^N vs. total N contents of *Arabidopsis thaliana* plants grown on a mixture of 0.75 mM 10 atom% excess U^15^N_2_U^13^C_5_-L-gln and 1.5 mM non-labelled NO_3_^-^.

**Fig. S1** Regression analysis of excess ^15^N vs. total N contents of *Arabidopsis thaliana* shoots (**a**) and roots (**b**) for plants grown on horizontal plates (not split root) on 10 atom% excess U^15^N_2_U^13^C_5_-L-gln. The theoretical slope in this experiment (provided correct mixing rate of 10 atom % ^15^N) is 0.1.


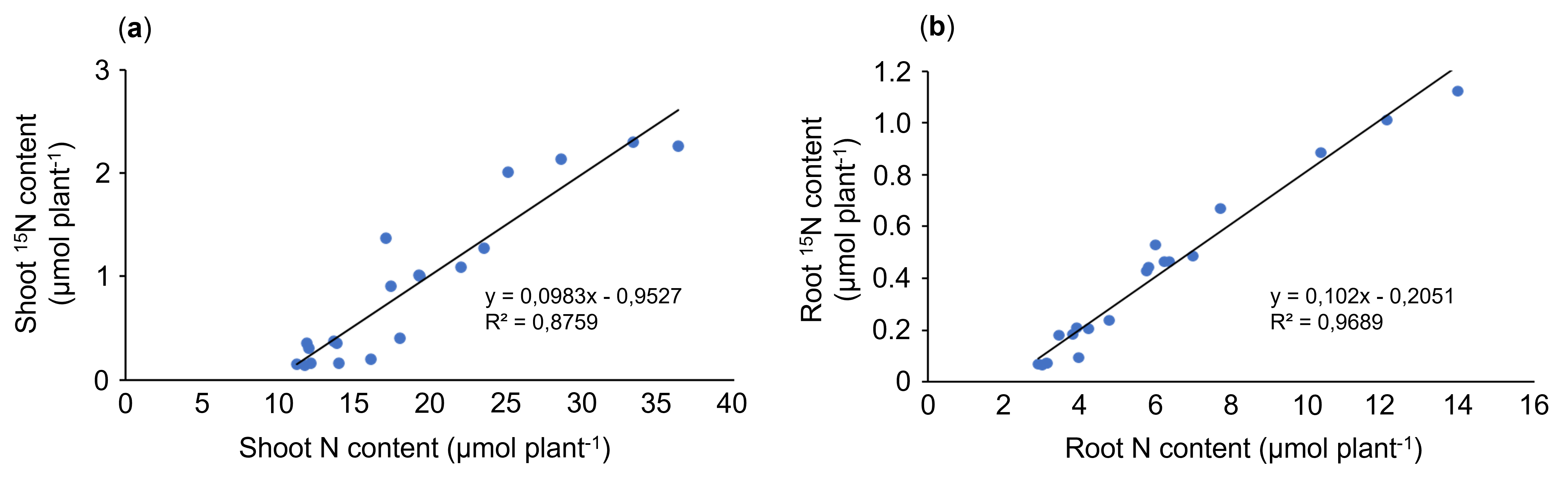


**Fig. S2** Regression analysis of excess ^15^N vs. total N contents in *Arabidopsis thaliana* shoots (**a**) and roots (**b**) of plants grown on horizontal plates (not split root) on a mixture of 0.75 mM 10 atom% excess U^15^N_2_U^13^C_5_-L-gln and 1.5 mM non-labelled NO_3_^-^. Dotted lines indicate theoretical regressions corresponding to either all plant N would have been derived from L-gln (upper dotted line) or 50% of plant N would have been derived from L-gln (lower dotted line).

**
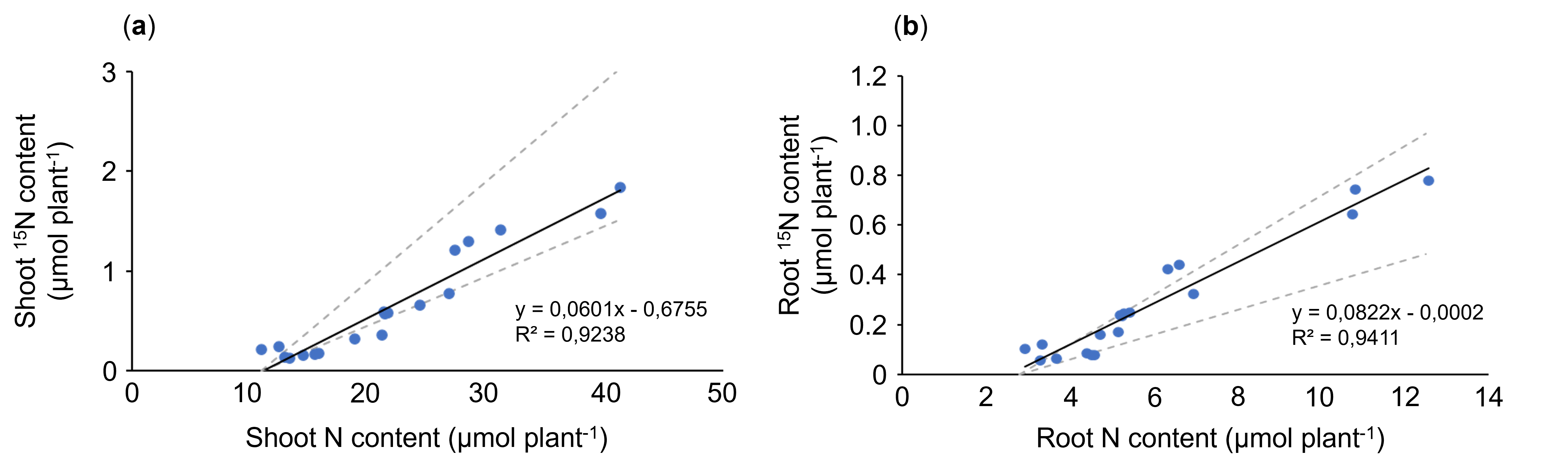
**
